# Cycle scheduling for in vitro fertilization with oral contraceptive pills versus oral estradiol valerate: a randomized, controlled trial

**DOI:** 10.1186/1477-7827-11-96

**Published:** 2013-09-28

**Authors:** Erik E Hauzman, Azucena Zapata, Alfonso Bermejo, Carlos Iglesias, Antonio Pellicer, Juan A Garcia-Velasco

**Affiliations:** 1 IVI Madrid, Avda del Talgo, 68-70, 28023 Madrid, Spain; 2 IVI Valencia, Plaza de la Policía Local, 3, 46015 Valencia, Spain; 3 Universidad de Valencia, Avda de Blasco Ibáñez, 13, 46010 Valencia, Spain; 4 Rey Juan Carlos University, Avda del Talgo 68-70, 28023 Madrid, Spain

**Keywords:** IVF, GnRH antagonist, Cycle scheduling, Oral contraceptives, Estrogen pretreatment

## Abstract

**Background:**

Both oral contraceptive pills (OCPs) and estradiol (E_2_) valerate have been used to schedule gonadotropin-releasing hormone (GnRH) antagonist in vitro fertilization (IVF) cycles and, consequently, laboratory activities. However, there are no studies comparing treatment outcomes directly between these two pretreatment methods. This randomized controlled trial was aimed at finding differences in ongoing pregnancy rates between GnRH antagonist IVF cycles scheduled with OCPs or E_2_ valerate.

**Methods:**

Between January and May 2012, one hundred consecutive patients (nonobese, regularly cycling women 18–38 years with normal day 3 hormone levels and <3 previous IVF/ICSI attempts) undergoing IVF with the GnRH antagonist protocol were randomized to either the OCP or E_2_ pretreatment arms, with no restrictions such as blocking or stratification. Authors involved in data collection and analysis were blinded to group assignment. Fifty patients received OCP (30 μg ethinyl E_2_/150 μg levonorgestrel) for 12–16 days from day 1 or 2, and stimulation was started 5 days after stopping OCP. Similarly, 50 patients received 4 mg/day oral E_2_ valerate from day 20 for 5–12 days, until the day before starting stimulation.

**Results:**

Pretreatment with OCP (mean±SD, 14.5±1.7 days) was significantly longer than with E_2_ (7.8±1.9 days). Stimulation and embryological characteristics were similar. Ongoing pregnancy rates (46.0% vs. 44.0%; risk difference, –2.0% [95% CI –21.2% to 17.3%]), as well as implantation (43.5% vs. 47.4%), clinical pregnancy (50.0% vs. 48.0%), clinical miscarriage (7.1% vs. 7.7%), and live birth (42.0% vs. 40.0%) rates were comparable between groups.

**Conclusions:**

This is the first study to directly compare these two methods of cycle scheduling in GnRH antagonist cycles. Our results fail to show statistically significant differences in ongoing pregnancy rates between pretreatment with OCP and E_2_ for IVF with the GnRH antagonist protocol. Although the study is limited by its sample size, our results may contribute to a future meta-analysis. An interesting future direction would be to extend our study to women with decreased ovarian reserve, as these are the patients in whom an increase in oocyte yield—due to the hypothetical beneficial effect of steroid pretreatment on follicular synchronization—could more easily be demonstrated.

**Trial registration:**

ClinicalTrials.gov http://NCT01501448.

## Background

In the last decade, gonadotropin-releasing hormone (GnRH) antagonist protocols have become increasingly popular for controlled ovarian hyperstimulation (COH). Their use offers several advantages over the long agonist protocol [1], including shorter overall treatment duration, absence of perimenopausal symptoms caused by pituitary desensitization, no risk of inadvertent administration at the beginning of pregnancy or of ovarian cyst formation, and a lower consumption of gonadotropins. According to the conclusions of a recent meta-analysis [2], similar live birth rates can be achieved with the use of GnRH antagonist protocols, while significantly reducing the risk of ovarian hyperstimulation syndrome, compared to the long GnRH agonist protocol.

A potential disadvantage of GnRH antagonist protocols is that, classically, stimulation is started on day 2 or 3 of the menstrual cycle, which makes it difficult to schedule stimulation and laboratory activities. Cycle scheduling can be used to avoid weekend retrievals and to equally distribute the workload throughout the week, thereby avoiding excessive opening of incubator doors and the associated negative impact on embryo development, as well as reducing the amount of unplanned work, which can result in loss of concentration and reduced efficiency of the laboratory staff [3-5].

Both oral contraceptive pills (OCPs) and synthetic progestogens have been used for many years to schedule cycles [6-9]. More recently, the use of natural estrogens has also been advocated [10]. Estrogens primarily inhibit FSH secretion, whereas progestogens are mainly involved in the control of LH secretion.

Some smaller-scale studies on the hypothetical negative impact of OCPs on IVF cycles yielded controversial results in terms of ongoing pregnancy rates [11,12], while a recent meta-analysis, which summarized the results of six randomized controlled trials, found that ongoing pregnancy rates in normal responders were affected by OCP pretreatment [13]. However, this meta-analysis was confounded by the use of different OCPs for a varying number of days and with a varying pill-free interval. As far as the underlying mechanisms are concerned, it has been postulated that the gestagen component of OCPs could exert a negative impact on endometrial receptivity in the subsequent cycle. Alternatively, low LH concentrations after OCP pretreatment could impair oocyte competence or endometrial receptivity when ovarian stimulation is performed with recombinant FSH without LH [14]. However, in a recent randomized, controlled trial performed by our group that used one type of OCP for a relatively narrow range of days followed by a fixed pill-free period, we did not find any significant differences in ongoing pregnancy rates or live birth rates compared to the long agonist protocol [15].

The use of oral estrogens started in the midluteal phase of the cycle preceding ovarian stimulation was proposed recently for cycle scheduling purposes, based on the inhibitory effect of estradiol (E_2_) on follicle growth through its negative feedback on the increase in FSH during the luteal–follicular transition [16], which stops as soon as E_2_ is discontinued. Pretreatment with estrogens offers the advantage of a shorter duration of pretreatment than OCPs. Previous studies showed that estrogen pretreatment does not affect cycle outcome as compared to no pretreatment [17-19] or to the long GnRH agonist protocol [20], while it does promote follicular growth coordination during ovarian stimulation [21]. However, none of the studies compared treatment outcomes directly with those obtained after scheduling with OCPs. The aim of our study was to see whether these two methods of cycle scheduling are equally effective in terms of ongoing pregnancy rate and the secondary outcome measures detailed below.

## Methods

### Subjects

**Figure 1:**
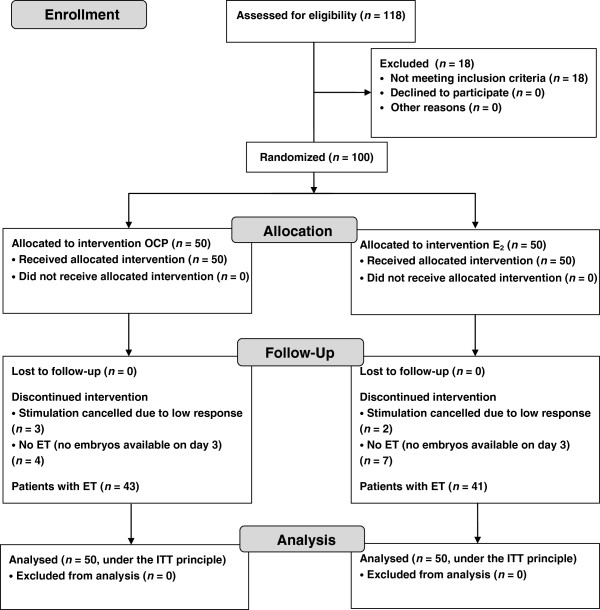
**CONSORT 2010 flowchart.** Note: ITT = intention-to-treat.

Between January and May 2012, we enrolled 118 women who were undergoing an IVF–ICSI cycle at IVI Madrid (Madrid, Spain). The inclusion criteria were age 18–38 years, regular normo-ovulatory menstrual cycles (26–35 days), body mass index (BMI) < 30 kg/m^2^, normal cycle day-3 basal serum hormone levels (FSH < 10 IU/l and E_2_ < 60 pg/ml), and <3 previous IVF/ICSI attempts. Exclusion criteria were previous ovarian surgery, low ovarian response (cancellation of the cycle due to poor follicular development after at least 7 days of gonadotropin stimulation, or <5 oocytes retrieved) in a previous IVF/ICSI cycle, and polycystic ovarian syndrome according to the Rotterdam criteria [22]. A total of 100 women were included in the study, with only one cycle per patient (Figure 1). This project was approved by our institutional review board, and all patients provided written informed consent. The study was registered with ClinicalTrials.gov (identifier: NCT01501448).

### Randomization procedure

A study nurse randomized and assigned patients at the time of cycle scheduling to either the OCP or E_2_ pretreatment arms in 1:1 ratio, based on a computer-generated random number list, with no restrictions such as blocking or stratification. The sequence was concealed in opaque, consecutively numbered envelopes until an intervention was assigned. Patients meeting all inclusion criteria were recruited consecutively. Authors involved in data collection and data analysis were blinded to group assignment.

### Protocols

Patients randomized to the OCP group started with the pill (30 μg of ethinyl E_2_ plus 150 μg of levonorgestrel [Microgynon; Bayer Schering Pharma, Berlin, Germany]) (*n* = 50) on day 1 or 2 of menses of the cycle prior to the scheduled IVF/ICSI procedure, and they took it for 12–16 days. As suggested by Cédrin-Durnerin et al. [23], ovarian stimulation was initiated 5 days after discontinuation of the pill, regardless of the specific day of the onset of menses, with a daily dose of recombinant FSH (Puregon; MSD, Oss, The Netherlands, or Gonal F; Merck Serono, Madrid, Spain) ranging from 200 to 225 IU on the first 5 days. From day 6 of stimulation, gonadotropin doses were adjusted according to serum E_2_ levels and ovarian response, which was assessed by vaginal ultrasound, every 2 days.

In patients allocated to the E_2_ arm of the study (*n* = 50), pretreatment with E_2_ valerate (Meriestra; Novartis Farmacéutica, Barcelona, Spain) was started on day 20 of the cycle preceding the IVF/ICSI cycle at a daily dose of 4 mg (2 mg twice a day) orally for 5–12 days, until the day before the initiation of ovarian stimulation, independent of the specific day of onset of menstruation, as it has been shown that the number of days by which E_2_ pretreatment is extended beyond the menses does not have a significant effect on cycle outcome [24].

The number of pretreatment days in both groups was chosen with the aim of starting stimulation between Wednesday and Sunday and thus equally distributing oocyte pickup among working days and minimizing weekend retrievals, as coordinated by a designated person at the centre.

In both groups, the GnRH antagonist ganirelix (Orgalutran; MSD) was introduced at a daily dose of 0.25 mg when the leading follicle reached 13 mm in mean diameter. Ovarian triggering was performed with 250 μg of recombinant human chorionic gonadotropin (hCG) (Ovitrelle; Merck Serono), which was administered as soon as two leading follicles reached ≥ 17 mm mean diameter. Ovum pickup was performed 36 hours later. IVF or ICSI was used to fertilize oocytes, according to individual requirements. A maximum of two embryos were transferred on day 3, under ultrasound guidance. Luteal support was started with 200 mg of micronized vaginal progesterone (Progeffik 200; Laboratorios Alcalá Farma, Alcalá de Henares, Spain) every 12 hours, beginning the night after ovum pickup. Serum βhCG was evaluated 12 days after embryo transfer; a result of > 10 IU/l was considered positive. Clinical pregnancy was confirmed by transvaginal ultrasound 2 weeks later if βhCG was positive.

Clinical pregnancy was defined as an intrauterine sac with heartbeat 4 weeks after embryo transfer, whereas ongoing pregnancies were characterized by the presence of a developing embryo at >12 weeks of gestation.

### Sample size estimate

The primary outcome of the study was ongoing pregnancy rate. Secondary outcome variables were implantation rate, clinical pregnancy rate, miscarriage rate, and live birth rate.

Sample size calculations were based on the results of our recently published previous study [15], in which ongoing pregnancy rates in GnRH antagonist cycles pretreated with OCP (47.8%) were compared with the GnRH agonist protocol. Based on a two-sided significance level of 0.05 and a power of 80%, at least 1,555 cycles in each group would have been necessary to detect a 5% difference in ongoing pregnancy rates. However, reaching a sample size large enough for an adequately powered investigation is not feasible for a single-centre study. Therefore, we arbitrarily chose the number of patients to be recruited in this study to provide clinically useful data that could be incorporated into a future meta-analysis.

### Statistical analysis

Categorical data are expressed as number and percentage, and continuous data as mean ± standard deviation (SD). For categorical variables, the χ^2^ test was used with continuity correction, whereas continuous variables were analyzed with Student’s *t*-test for independent samples, after testing for normality. A *P* value of <0.05 was considered to be statistically significant. All statistical analyses were performed with the SPSS 13.0 package (SPSS, Inc., Chicago, IL, USA).

## Results

As presented in Table 1, both groups of patients (*n* = 50 in both groups) were comparable in terms of age, BMI, and number of previous IVF attempts.

**Table 1:** Demographic characteristics of patients

**Characteristics**	**OCP (*n*** **= 50)**	**E**_**2 **_**(*n*** **= 50)**
*Age (years)*	33.9 ± 3.4	34.5 ± 3.1
*BMI (kg/m*^*2*^*)*	22.2 ± 3.6	20.9 ± 2.7
*Previous IVF/ICSI attempts*	0.5 ± 0.8	0.6 ± 0.9

Data are expressed as mean ± SD. There were no statistically significant differences as analyzed with Student’s t-test for independent samples.

Due to the difference between the two pretreatment protocols, significantly more days of pretreatment with OCP compared to E_2_ (14.5 ± 1.7 vs. 7.8 ± 1.9 days, *P* < 0.001) were necessary before starting stimulation. All parameters of stimulation that we analyzed (duration of stimulation, total amount of FSH used, and peak E_2_ and progesterone levels) were comparable between the groups (Table 2).

**Table 2:** Stimulation cycle parameters

**Characteristics**	**OCP (*n*** **= 50)**	**E**_**2 **_**(*n*** **= 50)**	***P *value**
*Pretreatment days*	14.5 ± 1.7	7.8 ± 1.9	<0.001
*Stimulation days*	10.0 ± 1.7	10.6 ± 1.5	0.09
*Total FSH dose (IU)*	1,627 ± 565	1,692 ± 488	0.54
*Peak E*_*2*_*(pg/ml)*	1,527 ± 729	1,596 ± 774	0.84
*Peak P (ng/ml)*	0.6 ± 0.3	0.5 ± 0.2	0.52
*Cancellation rate*	6% (3/50)	4% (2/50)	0.99
*Retrieved oocytes*	9.6 ± 4.9	10.2 ± 6.0	0.61
*Fertilization rate (%)*	64.0 ± 19.4	61.3 ± 20.8	0.52
*Top-quality embryos*	3.0 ± 2.2	3.0 ± 2.4	0.93
*Transferred embryos*	1.6 ± 0.4	1.4 ± 0.6	0.07

Continuous data are expressed as mean ± SD.

Stimulation was cancelled in three patients receiving OCP pretreatment and two patients pretreated with E_2_ due to low response in all cases. Of those patients who did undergo oocyte pickup (47 and 48 patients, respectively), four and seven women did not reach the stage of embryo transfer in the OCP and E_2_ groups, respectively, because no embryos were available on day 3 (Figure 1).

No significant differences were observed in the number of retrieved oocytes, fertilization rate, number of top-quality embryos, or number of transferred embryos. With regard to the outcome of the cycles, implantation rates, total and clinical pregnancy rates, early clinical miscarriage rates, and ongoing pregnancy rates and live birth rates were all comparable between the two groups (there were two late miscarriages in both groups) (Table 3). All analyses were based on the intention-to-treat principle. However, even an "as treated" analysis of the primary outcome measure, based on 23/43 vs. 22/41 ongoing pregnancies, would give a difference that remains far from statistical significance (*P* = 0.84).

**Table 3:** Treatment outcome parameters

**Parameters**	**OCP**	**E**_**2**_	**Risk difference (%) (95% CI)**	**P value**
	**(n = 50)**	**(n = 50)**		
*Implantation rate*^*a*^	43.5% (30/69)	47.4% (27/57)	3.9	0.79
			(-13.4 to 21.1)	
*Pregnancy rate per cycle*	56.0% (28/50)	52.0% (26/50)	-4.0	0.84
			(-23.1 to 15.4)	
*Clinical pregnancy rate per cycle*	50.0% (25/50)	48.0% (24/50)	-2.0	0.99
			(-21.3 to 17.4)	
*Early clinical miscarriage*^*b*^*rate per pregnancy*	7.1% (2/28)	7.7% (2/26)	0.6	0.66
			(-16.4 to 18.3)	
*Ongoing pregnancy rate per cycle*	46.0% (23/50)	44.0% (22/50)	-2.0	0.99
			(-21.2 to 17.3)	
*Live birth rate per cycle*	42.0% (21/50)	40.0% (20/50)	-2.0	0.99
			(-21.0 to 17.1)	

^a^Implantation rate is the number of intrauterine sacs with heartbeat / number of transferred embryos.

^b^Clinical pregnancies with miscarriage at <12 weeks of gestation.

*CI* confidence interval.

**Figure 2:**
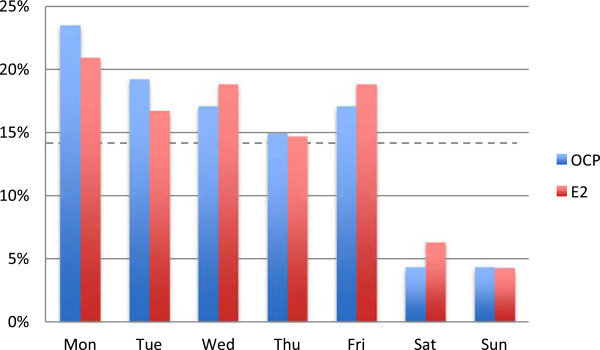
**Distribution of oocyte retrievals across days of the week with OCP and E**_**2 **_**pretreatment, respectively.** Note: Dashed horizontal line at 14.3% (1/7) shows proportion of retrievals per day expected to occur by pure chance.

The proportion of oocyte retrievals performed on weekend days was similar between the groups: 8.5% (4/47) with OCP and 10.4% (5/48) with E_2_ pretreatment (*P* = 0.97) (Figure 2). Both frequencies were significantly lower than 28.6%, which would be expected to occur by pure chance (i.e., on 2 out of 7 days) (*P* = 0.03 for OCP and *P* = 0.04 for E_2_).

## Discussion

Our results failed to show statistically significant differences for any measures of IVF treatment outcome between pretreatment with OCP and E_2_ for COH with the GnRH antagonist protocol. To the best of our knowledge, this is the first study to directly compare these two methods of cycle scheduling in GnRH antagonist cycles.

Cédrin-Durnerin et al. showed a significantly higher consumption of gonadotropins in OCP-pretreated cycles as compared to either pretreatment with E_2_ or no pretreatment, with no significant differences in treatment outcomes [23]. However, their study was mainly aimed at examining changes in the hormonal environment and antral follicles during the washout period after discontinuation of different methods of steroid pretreatment, and it included an even lower number of patients per treatment arm. Their data showed that steroid pretreatments differently affect the hormonal environment before the start of stimulation. According to their findings, it took 5 days after stopping OCP for FSH and LH to return to baseline levels from a strong suppression, suggesting this was an optimal washout period in cycles pretreated with OCP. In contrast, E_2_ pretreatment did not reduce FSH levels significantly but rather avoided the increase of FSH during the luteal-follicular transition, and the rapid FSH rebound after stopping natural estrogen intake argues for a short, 1–2 day washout interval. Indeed, we administered E_2_ until the day before the initiation of ovarian stimulation in order to optimize the synchronization of endogenous and exogenous FSH stimuli.

In a more recent study [19], the same group of researchers hypothesized that a 1-day washout period was too short to allow for complete recovery of baseline FSH levels, and that this was responsible for the increased gonadotropin consumption as compared to cycles without any pretreatment. However, no clinical studies have been performed with COH started 2 days after stopping E_2_ administration.

Although the length of steroid exposure varied considerably both within each pretreatment group and between the two groups, previous studies have shown that the number of pretreatment days – at least within the range of days used in our study – has no impact on COH outcomes in cycles pretreated with either E_2_[24] or OCPs [25]. There is, however, considerable difference between OCPs in terms of their suppressive effect, depending on their progestin component [26]. A recent, small-scale, retrospective cohort study, performed in egg donors, suggested that OCPs containing androgenic (estrane- and gonane-derived) progestins lead to a more profound suppression of follicular development than those with anti-androgenic activity, resulting in lower oocyte yields [27]. The authors hypothesized that OCPs including an androgenic progestin would inhibit gonadotropin support for the growing follicle but maintain androgen exposure, which would lead to initial androgen-driven follicle growth but, in the end, to atresia of growing follicles because of the lack of FSH support, whereas in patients treated with anti-androgenic OCPs, lacking the androgen-driven growth of small growing follicles and the growth support from FSH, very small follicles would fail to grow but would not undergo atresia either. Upon suspending OCPs, and starting COH, these small follicles would then still have the ability to resume growth and development, leading to ultimately larger oocyte yields than with androgenic HCs. However, this hypothesis, based on retrospective comparison of two relatively small groups, each receiving several types of OCPs, needs to be confirmed by prospective studies. In fact, these findings would not affect the results of our study, as we used a single type of progestin (levonorgestrel) in all our OCP-pretreated patients.

That weekend oocyte retrievals can be significantly reduced by using OCPs is well known [28]. Using this method ensures that there is no need to prolong the follicular phase by delaying administration of hCG, a practice that has been shown to result in lower pregnancy rates if triggering is delayed by 2 days [29]. However, as shown by Tremellen and Lane [30], a 1-day advancement or delay of hCG administration does not adversely impact IVF live birth outcomes. Earlier studies demonstrated that E_2_ pretreatment can also be used for optimal cycle scheduling by reducing weekend retrievals [18,24]. Moreover, extending E_2_ pretreatment beyond the menses has no deleterious effect on COH outcomes [24]. Our results are in line with these findings, as the proportion of oocyte retrievals performed on weekend days was significantly reduced using either method (Figure 2). Although the reduction in weekend retrievals was even more pronounced in a study by Blockeel et al. [18] (1/37 in the E_2_ pretreatment group vs. 8/39 in patients without steroid pretreatment), our aim was not to completely avoid weekend pickups, but rather to distribute pickups evenly among the days of the week, taking into consideration the lower number of personnel working on weekends. In fact, Blockeel et al. [18] initiated stimulation between Thursday to Sunday, whereas our practice is to start gonadotropin administration between Wednesday and Sunday. This one-day difference might account for the more pronounced reduction in weekend pickups in their study.

A major limitation of our study is its sample size. In fact, with 50 patients in each arm of the study, only a difference of >26% could have been detected with 80% power, at a 0.05 significance level. However, as mentioned earlier, our aim was to contribute our clinical results to a future meta-analysis on the subject. An interesting future direction would be to extend our study to women with decreased ovarian reserve, as these are the patients in whom an increase in oocyte yield—due to the hypothetical beneficial effect of steroid pretreatment on follicular synchronization—could more easily be demonstrated. As far as the use of OCPs in poor responders is concerned, a modification to the GnRH antagonist protocol has been proposed by Orvieto et al. [31] The so-called "ultrashort GnRH agonist/GnRH antagonist protocol" is supposed to combine the beneficial effects of OCP pretreatment with that of the gonadotropin flare induced by the GnRH agonist, which is administered at the beginning of COH. The latter could circumvent the alleged detrimental effect of the OCP pretreatment on endogenous LH levels, which in turn could impair oocyte competence or endometrial receptivity. This protocol has been used with success in poor responder patients [32]. However, no prospective studies have compared so far the efficacy of this combined approach to other stimulation protocols.

## Conclusions

In conclusion, we could not demonstrate differences in clinical results by using either OCP or E_2_ pretreatment for COH, if differences in washout periods after stopping pretreatment are accounted for, admitting, however, that the study was underpowered for the detection of small differences. Our results support those of earlier studies demonstrating that endogenous FSH suppression before starting ovarian stimulation is an efficient way to schedule ovarian stimulation in GnRH antagonist cycles. While observing comparable clinical outcomes, the use of E_2_ might offer several practical advantages compared to OCP: (1) Because E_2_ pretreatment is started in the midluteal phase, preparation for a programmed stimulation with a GnRH antagonist protocol can be scheduled in the same cycle even if the patient expresses a desire to start treatment after the early follicular phase. (2) Pretreatment is shorter with E_2_ than with OCPs (5–12 vs. 12–16 days, respectively, in our practice). (3) By using E_2_, GnRH antagonist cycles can be started in a scheduled manner even in patients who have objections to or present contraindications for taking OCPs even for a short period. Furthermore, by avoiding OCP pretreatment, we can give them one more chance to get pregnant spontaneously in the cycle preceding IVF.

## Competing interests

The authors declare that they have no competing interests.

## Authors’ contributions

The study was designed by AB, AP and JAGV. Data were collected by AZ and CI, analyzed and interpreted by EEH and JAGV. The manuscript was drafted by EEH and revised critically by all other authors. The study was supervised by AP and JAGV. All authors read and approved the final manuscript.

## References

[B1] Devroey P Aboulghar M Garcia-Velasco J Griesinger G Humaidan P Kolibianakis E Ledger W Tomás C Fauser BC Improving the patient’s experience of IVF/ICSI: a proposal for an ovarian stimulation protocol with GnRH antagonist co-treatment Hum Reprod 2009 24 764 774 19153090 10.1093/humrep/den468

[B2] Al-Inany HG Youssef MAFM Aboulghar M Broekmans F Sterrenburg M Smit J Abou-Setta AM Gonadotrophin-releasing hormone antagonists for assisted reproductive technology Cochrane Database Syst Rev 2011 5 CD001750 10.1002/14651858.CD001750.pub3 21563131

[B3] Mortimer D Mortimer ST Quality and risk management in the IVF laboratory 2005 Cambridge: Cambridge University Press

[B4] González RM Canales E García R Martín C Roldán M Fernández M Prados N Recuperación real de la temperatura y porcentaje de CO_2_ en los incubadores de fecundación in vitro Proceedings of the XXIth national congress of AETEL 2008 Madrid: AETEL

[B5] Janssens R Souffreau R Haentjens P Van de Velde H Verheyen G Clinical outcome after culturing human preimplantation embryos in incubators with individual chambers compared to standard incubators; randomised trial [abstract] Hum Reprod 2011 26 i40 i41

[B6] Frydman R Forman R Rainhorn JD Belaisch-Allart J Hazout A Testart J A new approach to follicular stimulation for in vitro fertilization: programmed oocyte retrieval Fertil Steril 1986 46 657 662 3758385 10.1016/s0015-0282(16)49644-5

[B7] Wardle PG Foster PA Mitchell JD McLaughlin EA Williams JAC Corrigan E Ray BD McDermott A Hull MG Norethisterone treatment to control timing of IVF cycle Hum Reprod 1986 1 455 457 3571438 10.1093/oxfordjournals.humrep.a136454

[B8] Zorn JR Boyer P Guichard A Never on a Sunday: programming for IVF-ET and GIFT Lancet 1987 1 8529 385 386 2880186 10.1016/s0140-6736(87)91756-9

[B9] Gerli S Remohí J Partrizio P Borrero C Balmaceda JP Silber SJ Asch RH Programming of ovarian stimulation with norethindrone acetate in IVF/GIFT cycles Hum Reprod 1989 4 746 748 2514191 10.1093/oxfordjournals.humrep.a136977

[B10] de Ziegler D Jääskelaïnen AS Brioschi PA Fanchin R Bulletti C Synchronisation of endogenous and exogenous FSH stimuli in controlled ovarian hyperstimulation (COH) Hum Reprod 1998 13 561 564 10.1093/humrep/13.3.561 9572410

[B11] Rombauts L Healy D Norman RJ Comparative randomized trial to assess the impact of oral contraceptive pretreatment on follicular growth and hormone profiles in GnRH antagonist-treated patients Hum Reprod 2006 13 235 245 10.1093/humrep/dei302 16253978

[B12] Kolibianakis EM Papanikolau EG Camus M Tournaye H Van Steirteghem AC Devroey P Effect of oral contraceptive pill pretreatment on ongoing pregnancy rates in patients stimulated with GnRH antagonists and recombinant FSH for IVF. A randomized controlled trial Hum Reprod 2006 21 352 357 16269449 10.1093/humrep/dei348

[B13] Griesinger G Kolibianakis EM Venetis C Diedrich K Tarlatzis B Oral contraceptive pretreatment significantly reduces ongoing pregnancy likelihood in gonadotropin-releasing hormone antagonist cycles: an updated meta-analysis Fertil Steril 2010 94 2382 2384 10.1016/j.fertnstert.2010.04.025 20537631

[B14] Griesinger G Venetis CA Marx T Diedrich K Tarlatzis BC Kolibianakis EM Oral contraceptive pill pretreatment in ovarian stimulation with GnRH antagonists for IVF: a systematic review and meta-analysis Fertil Steril 2008 90 1055 1063 10.1016/j.fertnstert.2007.07.1354 18054003

[B15] Garcia-Velasco JA Bermejo A Ruiz F Martínez Salazar J Requena A Pellicer A Cycle scheduling with oral contraceptive pills in the GnRH antagonist protocol vs the long protocol: a randomized, controlled trial Fertil Steril 2011 96 590 593 10.1016/j.fertnstert.2011.06.022 21718992

[B16] Le Nestour E Marraoui J Lahlou N Roger M de Ziegler D Bouchard P Role of estradiol in the rise in follicle-stimulating hormone levels during the luteal–follicular transition J Clin Endocrinol Metab 1993 77 439 442 10.1210/jc.77.2.439 8345049

[B17] Fanchin R Salomon L Castelo-Branco A Olivennes F Frydman N Frydman R Luteal estradiol pre-treatment coordinates follicular growth during controlled ovarian hyperstimulation with GnRH antagonists Hum Reprod 2003 18 2698 2703 10.1093/humrep/deg516 14645194

[B18] Blockeel C Engels S De Vos M Haentjens P Polyzos NP Stoop D Camus M Devroey P Oestradiol valerate pretreatment in GnRH-antagonist cycles: a randomized controlled trial Reprod Biomed Online 2012 24 272 280 10.1016/j.rbmo.2011.11.012 22296973

[B19] Cédrin-Durnerin I Guivarc’h-Levêque A Hugues JN Pretreatment with estrogen does not affect IVF-ICSI cycle outcome compared with no pretreatment in GnRH antagonist protocol: a prospective randomized trial Fertil Steril 2012 97 1359 1364 10.1016/j.fertnstert.2012.02.028 22464760

[B20] Ye H Huang GN Zeng PH Pei L IVF/ICSI outcomes between cycles with luteal estradiol (E2) pre-treatment before GnRH antagonist protocol and standard long agonist protocol: a prospective and randomized study J Assist Reprod Genet 2009 26 105 111 10.1007/s10815-009-9300-3 19225876 PMC2654939

[B21] Fanchin R Schönauer LM Cunha-Filho JS Méndez Lozano DH Frydman R Coordination of antral follicle growth: basis for innovative concepts of controlled ovarian hyperstimulation Semin Reprod Med 2005 23 354 362 10.1055/s-2005-923393 16317624

[B22] Rotterdam ESHRE/ASRM-Sponsored PCOS consensus workshop group Revised 2003 consensus on diagnostic criteria and long-term health risks related to polycystic ovary syndrome (PCOS) Hum Reprod 2004 19 41 47 14688154 10.1093/humrep/deh098

[B23] Cédrin-Durnerin I Bständig B Parneix I Bied-Damon V Avril C Decanter C Hugues JN Effects of oral contraceptive, synthetic progestogen or natural estrogen pre-treatments on the hormonal profile and the antral follicle cohort before GnRH antagonist protocol Hum Reprod 2007 22 109 116 16936304 10.1093/humrep/del340

[B24] Guivarc’h-Levêque A Homer L Arvis P Broux PL Moy L Priou G Vialard J Colleu D Dewailly D Programming in vitro fertilization retrievals during working days after a gonadotropin-releasing hormone antagonist protocol with estrogen pretreatment: does the length of exposure to estradiol impact on controlled ovarian hyperstimulation outcomes? Fertil Steril 2011 96 872 876 10.1016/j.fertnstert.2011.07.1138 21868004

[B25] van Heusden AM Fauser BC Residual ovarian activity during oral steroid contraception Hum Reprod Update 2002 8 345 358 10.1093/humupd/8.4.345 12206469

[B26] Phillips A Hahn DW Klimek S McGuire JL A comparison of the potencies and activities of progestogens used in contraceptives Contracept 1987 36 181 192 10.1016/0010-7824(87)90013-8 3427965

[B27] Barad DH Kim A Kubba H Weghofer A Gleicher N Does hormonal contraception prior to in vitro fertilization (IVF) negatively affect oocyte yields? - a pilot study Reprod Biol Endocrinol 2013 11 28 33 10.1186/1477-7827-11-28 23557032 PMC3637242

[B28] Barmat LI Chantilis SJ Hurst BS Dickey RP A randomized prospective trial comparing gonadotropin-releasing hormone (GnRH) antagonist/recombinant follicle-stimulating hormone (rFSH) versus GnRH-agonist/rFSH in women pretreated with oral contraceptives before in vitro fertilization Fertil Steril 2005 83 321 330 10.1016/j.fertnstert.2004.06.076 15705369

[B29] Kolibianakis EM Albano C Camus M Tournaye H Van Steirteghem A Devroey P Prolongation of follicular phase in in vitro fertilization results in a lower ongoing pregnancy rate in cycles stimulated with recombinant follicle- stimulating hormone and gonadotrophin-releasing hormone antagonists Fertil Steril 2004 82 102 107 15236997 10.1016/j.fertnstert.2004.01.027

[B30] Tremellen KP Lane M Avoidance of weekend oocyte retrievals during GnRH antagonist treatment by simple advancement or delay of hCG administration does not adversely affect IVF live birth outcomes Hum Reprod 2010 25 1219 1224 10.1093/humrep/deq059 20215127

[B31] Orvieto R Kruchkovich J Rabinson J Zohav E Anteby EY Meltcer S Ultrashort gonadotropin-releasing hormone agonist combined with flexible multidose gonadotropin-releasing hormone antagonist for poor responders in in vitro fertilization/embryo transfer programs Fertil Steril 2008 90 228 230 10.1016/j.fertnstert.2007.06.022 17681292

[B32] Orvieto R The ultrashort flare GnRH-agonist/GnRH-antagonist protocol enables cycle programming and may overcome the "detrimental effect" of the oral contraceptive Fertil Steril 2012 98 e17 18 10.1016/j.fertnstert.2012.06.053 22840381

